# Costs and Arising Work Times of Volatile Short-Term Sedation in Intensive Care

**DOI:** 10.3390/healthcare13141732

**Published:** 2025-07-18

**Authors:** Armin Niklas Flinspach, Michaela Pfaff, Florian Jürgen Raimann

**Affiliations:** Department of Anaesthesiology, Intensive Care Medicine and Pain Therapy, University Hospital Frankfurt, Goethe-University Frankfurt, Theodor-Stern Kai 7, 60590 Frankfurt/Main, Germany

**Keywords:** volatile sedation, management, awakening, delirium, economics, financial disclosure, financial management

## Abstract

**Background**: Optimizing critical care sedation is an important and complex task. Although intravenous sedatives are widely used, they do have limitations compared to volatile sedatives, such as faster awakening and minimal accumulation. However, volatiles are still rarely used due to technical barriers and costs. We intended to conduct an economic evaluation on the workload and efficiency of short-term volatile sedation. **Methods**: Retrospective secondary data analysis of the cost of 60 min sedation after cardiac valve surgery performed at a tertiary center (n = 94), including assessment of material turnover, substance consumption and personnel expenses combined on a monetary basis. **Results**: The time required for bedside preparation was extended from almost 18 min (i.v. sedation) to an additional 9–10 min when applying volatile sedatives. We calculated a median sevoflurane consumption of 23 mL using MIRUS^TM^ and 14 mL using Sedaconda, resulting in an average price of EUR 38.43 for propofol, EUR 13.24 for sevoflurane under Sedaconda, and EUR 15.03 using MIRUS^TM^ for application in the monetary evaluation. The total prices were calculated based on an additionally optimized scenario of weekly use of a MIRUS^TM^ reflection device system, at EUR 128.99 versus EUR 119.73 (Sedaconda) versus EUR 48.44 for conventional propofol-based sedation. **Conclusions**: The use of volatile sedation in intensive care has a higher price in short-term use due to the cost of the single-use reflector of the anesthetic conserving device, which is difficult to offset financially against the pharmacological benefits in terms of faster recovery. However, the additional setup times are relatively short. Clinical benefits such as faster recovery were not included in the cost analysis.

## 1. Introduction

In recent decades, there has been an increasing focus on optimizing intensive care sedation procedures, particularly based on the emerging evidence of reduced mortality under minimal to zero pharmacological sedation [1,2,3]. Therefore, the question of suitable procedures is all the more complex when (short-term) deep sedation is required.

However, after benzodiazepines were found to have outcome-relevant side effects, particularly an increased incidence of delirium, a paradigm change took place in the early 2000s, and the use of these agents was explicitly discouraged in guidelines [4,5]. Among the remaining intravenous sedatives, propofol and dexmedetomidine were henceforth used [6].

Volatile sedatives were rarely used in intensive care medicine despite their known potentially beneficial pharmacological profile, which is why they are not yet included in any Enhanced Recovery After Surgery (ERAS) program, for example from the Cardiac Society [7]. This may be due to the relatively short presence on the medical device market and low market penetration as a result of high acquisition cost (first Anaesthetic Conserving Devices (ACD) since 2004, of AnaConDa (renamed in 2021 to Sedaconda) (Sedana Medical AB, Danderyd, Schweden) and since 2013, of MIRUS^TM^ (Technologie Institut Medizin GmbH, Tübingen, Germany)) and initial focus initially primarily on the European market [8]. The possibility of using volatile sedatives on an intensive care ventilator via a reflective membrane (such as Sedaconda or MIRUS^TM^) with a reduced substance turnover (70–80% reflection rate) has opened up a much wider range of applications. Volatile sedatives are distinguished through their lack of accumulation and metabolization, which enabled significantly accelerated awakening and extubation [9,10,11]. Whether there is a reduction in the incidence of delirium or even mortality in critically ill patients appears to be doubtful at present [10]. However, the aggravated sedation of COVID-19 patients and the launch of MIRUS^TM^ appear to have led to more widespread use of the application in intensive care units (ICU) [12,13,14].

However, there is still a reluctance to use reflection devices on a large scale, motivated by the additional setup effort required, as well as the fear of additional prices despite proof of economic equivalence [15]. Improvements in the database of digital documentation as well as cost increases in almost all healthcare systems worldwide are driving the increased interest in monetary analysis [16,17]. These analyses are increasingly being used to assess the potential monetary benefits of customized situations after the early inventors’ phase has ended and the first studies are available, with the aim of selecting the methodology that will deliver rapid profitability from the wide range of new methodologies available.

To date, it is unclear how to quantify in detail the costs and arising work times associated with volatile sedation compared to propofol, as well as a comparison of the different devices.

**Figure 1:**
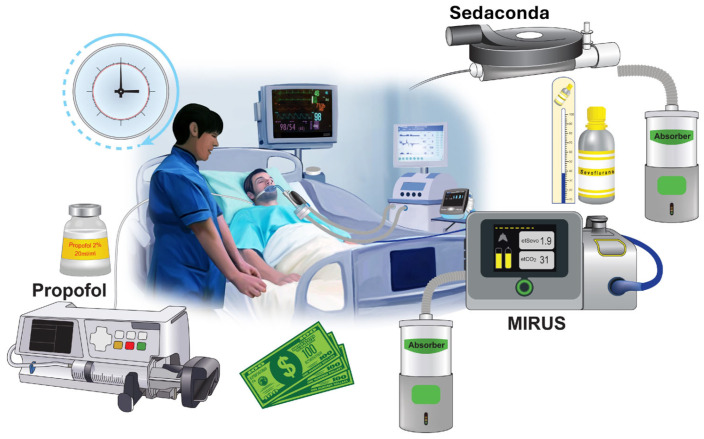
Volatile sedation economic assessment. The figure is an illustration of the economic correlations of sedation management with different substance classes and the associated setup and dismantling costs as well as the required personnel deployment in ongoing operations. The illustration shows the three sedation alternatives examined, propofol (bottom left) with syringe infusion pump, and the MIRUS^TM^ device with absorption system (right bottom). MIRUS^TM^ and the AnaCoNDa/Sedaconda system (top right) both use sevoflurane as a volatile substance to maintain sedation in the intensive care patient shown, while the illustrated labor time (top left) is also relevant to the study.

We therefore intended to use a prospective study data set to carry out a corresponding analysis of the various methodologies and work measures used (illustrated in Figure 1).

## 2. Materials and Methods

Based on a previously published study investigating the short-term use of volatile sedation in 94 patients undergoing heart valve surgery, a secondary data analysis was carried out with regard to the financial evaluation of expenditure on working time [18,19]. The initial study protocol was approved by the institutional ethics committee (#20-1050) and registered at clinicaltrials.gov on 1 July 2021 (NCT04958668) before the first patient enrollment. An informed consent was obtained from each individual patient or their legal representative in advance. The study was conducted at a tertiary care provider with a large department for cardiac surgery where patients are treated in a corresponding interdisciplinary intensive care unit. The intensive care team was familiar with both devices used in the trial. The study was conducted in Germany, where health insurance is compulsory so that every resident has access to full medical care.

### 2.1. Patient Population

Within the study period of 22 months, post-cardiac valve surgery patients underwent block randomization in order to be assigned to the treatment groups (volatile sedation or intravenous sedation management) and to receive the intended 60 min of post-procedure observation according to the study protocol. A critical evaluation of the patient’s condition was performed before the end of sedation, and consequent extubation was performed. The design of the initial study allowed a precise comparison of the workload during this time interval due to the strict specification of 60 min of sedation. The time interval was chosen based on the necessary clinical evaluations, in terms of the initial laboratory results, and the expected time to clinical stabilization of the patient’s condition, as opposed to the desire to keep the time under sedation and ventilation as short as possible in accordance with the ABCDE bundle guidelines. Minor deviations from the intended 60 min were caused in some cases due to delayed laboratory results, including blood gas samples and internal consultations, despite sufficient stabilization of the patient’s condition. The time recording was carried out by ANF or MP as separately trained staff. In addition to monetary quantification, the secondary data analysis also aimed to compare the anesthetic conserving devices (MIRUS™ Reflektor, a manufacturer’s technical update to Lisa44 has been available since 2023) and Sedaconda-S with 50 mL of dead space, commercially available due to the fact that both devices were used in the initial study.

### 2.2. Study Protocol

In order to determine the prices incurred, single-use and multiple-use material required for sedation application was assessed in terms of syringes, supply lines, absorbers, and filters. For this purpose, the patient-specific requirements for sedation were recorded with a subsequent monetary valuation in Euro. The times required to set up the bedside for the care of a patient undergoing cardiac surgery and any additional setup and dismantling times for sedation using volatile sedatives were also recorded, and the preparation was measured. Setup time for volatile sedation included preparing the Sedaconda syringe and dosing pump, as well as tubing and volatile gas monitoring setup. For MIRUS, this included the electrical setup, such as checking the tank fill level and refilling if necessary, as well as entering the required data into the electrical devices. The time required for setting up the absorber, including the tubing, was also recorded. Dismantling time included the orderly removal of relevant components from the bed space and, where necessary, the appropriate disposal of waste. Staff costs were calculated based on the largest existing public pay agreement for nursing staff (covering around 1.6 million employees in Germany) and the corresponding weekly 40 h work requirement. To estimate the costs incurred, an experienced intensive care nurse of average age with 10 years of professional experience was determined without adjusting the average working hours due to holiday entitlements or sick leave. Nevertheless, it must be considered that salaries can fluctuate slightly between individual collective agreements and that the individual age structure of the employee pool has an impact on salary categorization as well as possible sickness-related absences.

Furthermore, a financial evaluation of the required hardware was carried out to quantify the required investment, specifically the MIRUS^TM^, continuous anesthetic gas monitoring (e.g., Treaton; Sedana Medical AB, Danderyd, Schweden), absorber scales (CONTRAfluranTM, Zeosys Medical GmbH, Luckenwalde, Germany), and syringe infusion pump (e.g., Perfusor Space; B.Braun, Melsunge, Germany). In order to calculate the costs regardless of the required investments, a corresponding calculation was made of the substance quantities required for sedation and the associated costs. In order to financially quantify the working hours determined on the bedside using a timer, the costs were calculated on the basis of the applicable collective labor agreements.

The costs of the required sedatives were calculated based on the average costs of the four major manufacturers of propofol, as well as the prices of the six available manufacturers of sevoflurane (last evaluation in December 2024). The prices compared refer to the cost-intensive pharmacological price structure of the Federal Republic of Germany.

Within the study, patients were monitored from the beginning of the operative procedure until discharge from the ICU, in particular, to determine possible laboratory chemical effects (heart, kidney) and neurocognitive alterations (delirium).

### 2.3. Statistics

Categorical variables are presented as counts and percentages. Not normally distributed variables are described as medians (interquartile range, IQR 25/75). Demographics and clinical differences between groups were assessed using Fisher’s exact test for categorial variables and Mann–Whitney U-test as well as Kruskal–Wallis test and two-sided *t*-test for continuous variables, as appropriate. All statistical tests were two-tailed, and results with *p* < 0.05 were considered statistically significant. All analyses were performed with SPSS (IBM Corp., Version 29, Chicago, IL, USA).

## 3. Results

The complete data sets of 94 sedation episodes with an intended duration of 60 min (median 67 min (61/92)) were collected, of which 47 patients were sedated with propofol, 23 sedation episodes were performed using the reflection device MIRUS^TM^, and a further 24 using the Sedaconda system (Table 1).

There were no differences in terms of demographic (BMI, age, previous illnesses) or surgical characteristics (duration of surgery, time on CPB, duration of aortic clamping) between intravenous and volatile sedation. There was no difference in application time between MIRUS^TM^ and Sedaconda (66 ± 7 min vs. 65 ± 10 min; *p* = 0.91), nor in eye opening (83 ± 26 min vs. 89 ± 23 min; *p* = 0.58), hand squeezing (91 ± 23 min vs. 95 ± 22 min; *p* = 0.59), or time to extubation (92 ± 23 min vs. 92 ± 22 min; *p* = 0.95).

### 3.1. Personnel Expenses

With regard to the preliminary setup of the individual environment for the care of a complex post-cardiac surgery patient, the median required working time of an intensive care nurse was 17 min and 57 s (IQR 12:14/22:14). There were no differences in setup times between the various procedures with regard to the primary bed position for the care of a post-operative cardiac surgery patient. Additional time was required to explicitly set up the bed for volatile sedation, a median of 10 min and 30 s (IQR 08:23/14:20, [95% CI, 10:04–14:00]) for the MIRUS^TM^ system, and a median of 9 min and 23 s (IQR 08:14/13:11, [95% CI, 9:39–12:36]) for the Sedaconda system, without a statistically significant difference between the two volatile devices (*p* = 0.51). The same was shown for the necessary time for dismantling the required equipment (*p* = 0.15). The monetary calculation in accordance with the standard contractual terms amounted to EUR 10.01 in labor costs for the regular preparation of the bed site and EUR 17.69 for Sedaconda or EUR 19.57 for MIRUS^TM^ for preparation of the bed site, setup, and dismantling.

### 3.2. Substance Expenses

**Figure 2:**
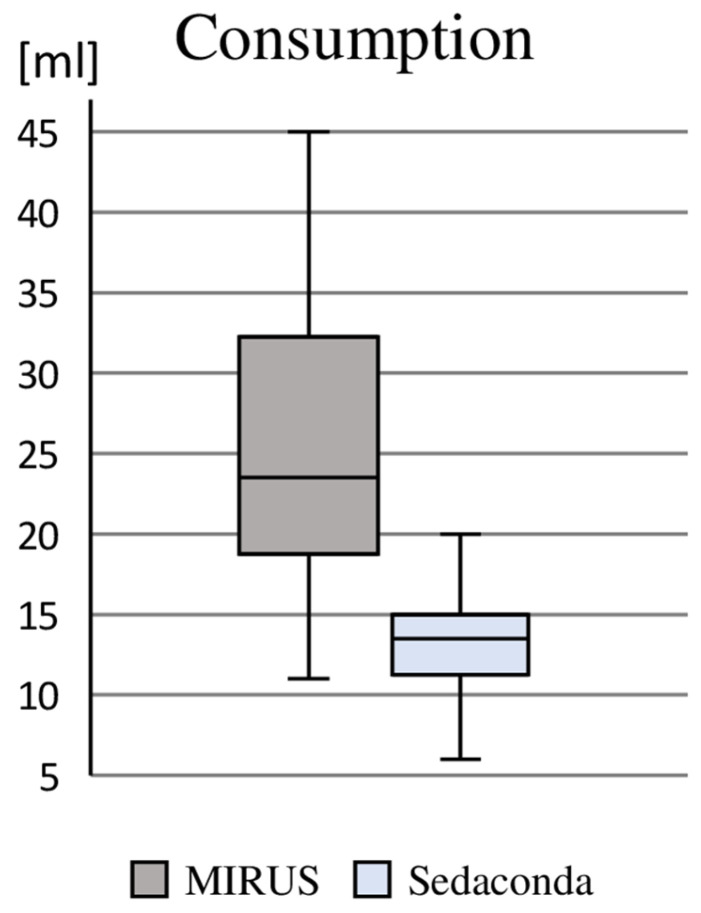
Consumption of sevoflurane. Reproduction of the sevoflurane consumption to maintain a hour of sedation without consideration for the wastage occurring under AnaConDa/Sedaconda.

Based on the use of 60 min, the majority of patients required one syringe of propofol 2% (containing 50 mL), and an increased requirement necessitated the use of one (n = 17) or two (n = 2) additional syringes; this corresponded to a median consumption of 386 mg (275 mg/739 mg). However, the delivery of 50 mL vials of 1000 mg (prepared in syringes at the bedside) resulted in the consumption of 66 vials within the intravenously sedated group (average wastage of 28 mL propofol 2% ≙ 560 mg). With regard to the consumption of sevoflurane, we found a median demand of 23 mL (IQR: 19 mL/32 mL) using the MIRUS^TM^ and 14 mL (IQR: 12 mL/15 mL) using Sedaconda, whereby, due to the prior sampling of sevoflurane into a special syringe, 486 mL had to be used for the 24 patients sedated with Sedaconda (average wastage of 7 mL sevoflurane), whereas no wastage occurred with the MIRUS^TM^ system (Figure 2).

With regard to the monetary evaluation, the average substance prices were EUR 38.43 for propofol 2% (average purchase prices in 2024 of the four available manufacturers), EUR 13.24 for sevoflurane under Sedaconda plus EUR 4.81 for disposable material for application (480 mL total consumption for 24 patients), and EUR 15.03 under MIRUS^TM^ (511 mL total consumption for 23 patients).

### 3.3. Material Expenditure

We found a static consumption of disposable products in our cohort, with differences within the sedation groups regarding the prices for intravenous sedation of EUR 4.80 (syringe, supply line, flashback valve) and due to the requirement of an additional draining tube system and an absorber as well as the differences in the purchase price of the reflection device amounting to approximately EUR 144.00 for Sedaconda and EUR 395.00 for MIRUS^TM^. The reusability of the drainage tube system and the absorber (complete filling) allows for optimization, so that the material prices for volatile sedation in our cohort were EUR 89.10 for Sedaconda and EUR 332.10 for MIRUS^TM^. The special feature of the MIRUS^TM^ reflection device with exchangeable Heat-Moist-Exchange (HME)/antibacterial filter enables the reflection device to be used for multiple patients within 1 week in accordance with the approval. Assuming daily use for one patient (five in total), the material prices are thus reduced to EUR 97.30 for the individual application, taking into account the additional HME.

### 3.4. Investments

Establishing volatile sedation requires the fulfillment of additional technical requirements in addition to the syringe pumps regularly available (investment prices of around EUR 5000 per device depending on manufacturer and purchase quantity). While the MIRUS^TM^, which is priced at around EUR 20,000, includes an anesthetic gas measurement system featuring integrated delivery mechanisms for closed-loop sedation, Sedaconda requires a separate anesthetic gas monitoring system and a syringe pump with special syringe for application. With regard to anesthetic gas monitoring, there are various applicants, which vary considerably in price depending on the functionality (e.g., integrated calorimetry), starting around EUR 4000. To optimize the use of absorbers, there are systems for detecting the fill level, which incur additional costs.

**Figure 3:**
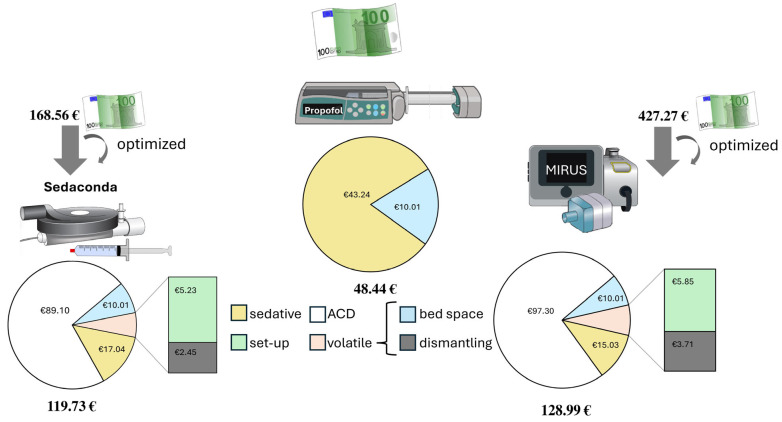
Total expenditure. Pie chart illustration of the material and personnel cost shares for one-hour sedation under optimized conditions. The illustration shows the three methods analyzed: Sedaconda, reflection device -S with syringe on the left, propofol for intravenous sedation via tip pump in the center, and MIRUS^TM^ with reflector membrane on the right. The initial price calculations included the costs for a one-time setup, including all required materials, pharmaceuticals, and absorption technology and tubing systems, as well as the calculation of a nurse’s working time for this setup work and dismantling time. An optimization of the cost structure was calculated on the basis of the fact that the absorption technique can be used several times, and the reflector membrane of the MIRUS^TM^ system can be used for several patients. The costs itemized in the pie chart include the single-use material costs and substance turnover, as well as personnel costs for setup and dismantling. For the reflector membrane, a single use was assumed for the Sedana Medical Device and a five-fold use for the MIRUS^TM^/LISA-44 Device. Abbreviation: reflection device, anesthetic conserving/reflection device, assumed price of the reflector system (Sedaconda or MIRUS^TM^).

The concrete total costs, excluding investment or leasing prices, resulted in EUR 168.56 (Sedaconda) versus EUR 427.27 (MIRUS^TM^) versus EUR 48.44 (propofol) for 60 min short-term use. Assuming that the hose system (Fluabsorb Accessory Kit, EUR 25.00) and absorber (Fluabsorber, EUR 50.00) may be used several times until full absorption capacity, the prices declined to EUR 119.73 (Sedaconda) versus EUR 359.09 (MIRUS^TM^) versus EUR 54.13 (propofol). The additionally optimized assumption of a weekly possible use of a MIRUS^TM^ reflection device system on five different patients resulted in EUR 119.73 (Sedaconda) versus EUR 128.99 (MIRUS^TM^) versus EUR 48.44 for conventional propofol-based sedation (see Figure 3).

## 4. Discussion

Our study aimed to determine the additional financial costs and labor requirements of volatile sedation administered to critical patients. In our retrospective analysis, we were able to detect substantial additional costs for the use of volatile sedation, which, due to the considerable additional costs of the reflection device itself, could be attributed to the short-term use and the equally necessary personnel expenses for setup and dismantling.

Volatile sedation has experienced a considerable boost in critical care in recent years; in particular, the excellent controllability and the possibility of deep sedation under COVID-19 brought an increasing spread within numerous European countries [12,13,14,20]. However, the feasibility of comprehensive volatile sedation in the ICU setting requires a closer look at the underlying healthcare system and the invoicing fundamentals. If payments are made for a specific healthcare service, such as a heart valve replacement, regardless of the actual effort involved, a faster transfer from the intensive care unit (ICU) can result in additional surgical turnover. However, this can be prevented by blocking factors, such as those in the German-DRG system, where intensive therapy is only eligible for billing after at least 24 h of hospitalization. The diagnosis-related group (DRG) system classifies hospital cases into groups for billing. Each group has a fixed reimbursement rate, encouraging cost-efficiency. Hospitals are paid a set amount per case, not per service, affecting billing by standardizing payments regardless of actual care costs.

The use of volatile sedation leads to a considerably earlier awakening and faster neurological examination. The evidence for accelerated wake-up times after short-term critical care sedation may be difficult to prove, depending on the benzodiazepines (midazolam) often used for comparison and the lack of dose control (e.g., target controlled infusion (TCI) according to the Marsh model for propofol), as was the case in our study. Especially with very short sedation under intensive supervision and the use of easily controllable analgesics such as remifentanil, the difference in wake-up times could be relativized. Even with optimized use of resources, using volatiles leads to a quantifiable increase in prices according to our data collection. This significant increase in costs of more than 240% plus any existing investments under sevoflurane sedation appears to be an understandable obstacle to widespread use. However, extending the sedation period to 2–24 h would primarily incur additional pharmaceutical costs, which should be considered if longer periods of sedation are desired. Similarly, with regard to the continuation of post-operative sedation after transport of the patient, e.g., in the theatre, it should be borne in mind that propofol syringes generally provide a substance volume for several hours and that, under certain circumstances, a short post-operative intensive sedation may be possible without a new syringe and thus without further costs. However, advantages that are difficult to quantify, such as shorter length of ICU stay seen in this cohort (*p* = 0.078), the shortened sedation time with regard to delirium risk, bronchodilator and anticonvulsant properties, and shortened ventilation time must also be taken into account, as these aspects can lead to increased indirect costs [19,21,22]. The assumable benefits include shortened intensive care stays with presumably lower mortality rates, a shortening of the length of time invasive catheters and drains remain in place, as well as earlier mobilization. The incidence of delirium and post-intensive care syndrome may also be reduced.

The optimal sedation regime in intensive care medicine as well as in cardio anesthesia has been the subject of an emotional debate for years [23,24]. While ultra-short-acting, context-insensitive opioids with the substance remifentanil are already available, as well as highly potent induction opioids such as sufentanile, the situation with sedatives is different [25,26]. Each substance has its own disadvantages and advantages, ranging from pharmacokinetic and pharmacodynamic profiles to long-term usability and ecological aspects. In principle, volatile sedatives are limited by their way of application and the resulting technical requirements. The substance-specific advantages must also be brought at the cost of a significant increase in material costs, particularly in short-term use. Measured against the operating and supply prices of an intensive care bed, sums of EUR 70 for starting and maintaining 60 min of sedation do not appear particularly relevant, but cannot be reduced any further without other benefits having to compensate for this. In particular, it should be noted that an extension of the sedation to more than 60 min only increases the substance-specific costs, up to a maximum application time of 24 h. In the case of the MIRUS^TM^ system, after 24 h, a change of the cost-effective HME is required, and in the case of the Sedaconda, it is required that the reflection device be changed. However, this results in considerable savings if a sedation period of more than 60 min is required.

The additional optimization of the devices presented results in a significant reduction in the cost of MIRUS, based on its assumed use with multiple patients. While the assumption that the MIRUS reflection device could be used with five patients was theoretical, it could be applied in individual cases in the underlying primary clinical study and nevertheless corresponds to clinical reality. Due to the study design and the fact that not all patients were continuously sedated with the corresponding device, implementation was limited to a scenario involving five patients. The choice of five patients for a reflection device that can be used for up to 7 days assumed that it would only be used for elective weekday procedures.

The prices of pharmaceuticals we have determined refer to the German market. This is particularly relevant given that, in international comparison, the costs of generically available pharmaceuticals may only amount to a fraction of the prices in Germany. For example, the price of propofol in Belgium is only 22% of the price of the product in Germany. This fact underlines the role of reflector membranes as the main cost factor in terms of the international transferability of our results.

We observed an interesting difference in the amount of substance required between the two volatile techniques. This could be due to the properties of the absorbers, the technical design of the devices, or the absorber surface to be saturated initially, which could be important in a short-term application. Differentiated reflection properties have already been reported [27]. With regard to the cost structure of the application of volatile sedatives, it must be taken into account that both the MIRUS^TM^ terminal device and an anesthetic gas measuring device regularly used for Sedaconda application are associated with considerable investment costs.

Volatile sedatives are all ecologically problematic polyfluorinated alkyl (PFA) substances, some of which have considerable global warming potential [28]. Even if efforts are being made to develop absorption (as we did and calculated) and closed-loop recycling, these substances remain problematic [29]. Their use in intensive care sedation remains associated with a high consumption of volatile sedatives, even with increasing improvements in reflection properties. Even if correct use results in low levels of contamination, sufficient air circulation in the ICU should be ensured in order to minimize the exposure of the practitioners [30]. Sevoflurane has been discarded after a single draw-up; in accordance with the legislative and microbiological requirements, re-filling into storage containers might be conceivable in individual countries (actually prohibited in Germany). This could be important from a health economic and ecological point of view. Optimizing the resulting waste could be achieved through more direct dialogue about the volatile anesthesia requirements during surgery, enabling a patient-specific volume to be determined to minimize waste.

Although data exist that suggest that a large percentage of European ICUs are basically equipped with the corresponding technology, widespread use would require the provision of a significantly larger number [12,13]. In the cost calculation presented, we excluded the acquisition prices, as MIRUS^TM^ can now be obtained from the manufacturer on a leasing basis for a fee based on hours of use, and many intensive care units have the technical prerequisites for detecting volatile sedatives (anesthetic gas measuring device) as an integrated component of the existing capnography. A comparison with the use of intravenous sedatives without therapeutic drug monitoring in clinical practice, and thus measured on the basis of the Richmond Agitation and Sedation Score (RASS), strongly suggests this.

With regard to the aspect of setup and dismantling times, it should be taken into account that the establishment of fixed application-specific bed spaces could level out the costs incurred here. Insofar as the absorber technology and the MIRUS^TM^ or anesthetic gas measuring device already exist permanently at individual bed locations, the EUR 7.68 and EUR 9.56 in required working time would be eliminated.

The consideration of additional working time as a solely monetary time component is not unproblematic, given that work services are actually stepped costs and therefore may be modeled in a more complex manner. In clinical practice, however, these additional work steps are not performed by additional staff, but are included as part of bedside preparation. In line with the intention to quantify this additional work, we did not model the virtual additional staff requirements. Overall, personnel costs are rather low. Although there are additional costs on top of EUR 10.01, these are less than EUR 10 in both techniques and can therefore be considered minor. Similarly, the difference in personnel costs between the volatile devices is only EUR 1.88 (corresponding to an additional expense of 24.5%), which is fairly insignificant compared to the substance and material costs. In addition, the study was conducted in a tertiary cardiac surgery ICU that was already familiar with volatile anesthetics. In accordance with applicable legal requirements at the study center, the ICU had a staffing ratio of one nurse to two patients. Deviations from this ratio could seriously compromise the feasibility of a well-structured setup and dismantling process due to interruptions in order to provide adequate care for other patients. Depending on the expertise of the nursing team in charge, it can also be assumed that physician staff resources would be required to a considerable extent to operate the equipment, which could result in significant additional costs based purely on standard wage rates.

In conclusion, it might be summarized that the additional costs of volatile short-term sedation of 60 min described in our study are presumably reduced over time; this should be investigated further in a structured manner. The advantages of volatile sedation include the effects of optimal sedation control with rapid awakening, shortened ventilation time and possibly reduced length of ICU stay, as already known in both our study and previous observations.

### Limitations

Due to the limited cohort of only 94 patients and the underlying single center approach, we have to accept several limitations in this study. Prices of pharmaceuticals are subject to substantial fluctuations, as COVID-19 has recently shown, and can be significantly influenced by purchasing strategies such as buying cooperatives, which we were unable to take into account. There are also significant price differences between countries. In Germany in particular, the retail prices of pharmaceuticals are high by European standards. This may have a significant influence on the differences in the prices to be paid for sedation pharmaceuticals. Despite the sample size, there may be personnel-specific fluctuations in the build-up speed, which we were unable to record. This includes the fact that younger employees incur lower costs and are more open to new technologies and methodologies than more senior team members. Likewise, costs that may result from prolonged sedation, such as delirium or complications of prolonged ventilation, are difficult to measure or calculate in monetary terms.

## 5. Conclusions

We were able to show that the short-term use of volatile sedatives is associated with considerable additional prices compared to intravenous sedation with propofol using the two anesthetic conserving devices available. The labor costs for setup as well as dismantling at the bedside only played a minor role. The reflector, in particular, caused the significant additional costs, which persisted even in the case of optimized use (multiple use where possible) but may be reduced if used for more than the 60 min sedation observed here.

## Figures and Tables

**Table 1:** Patient characteristics.

	Sedaconda	MIRUS^TM^	Intravenous	Overall
*n* =	24 (26.6%)	23 (24.4%)	47 (50.0%)	94 (100%)
Sex (male, %)	16 (66.7%)	17 (73.9%)	33 (70.2%)	66 (70.2%)
Age (years)	62 (49; 70)	60 (54; 67)	64 (57; 71)	62 (54; 71)
BMI (kg/m^2^)	26.5 (23.8; 30.8)	25.9 (23.4; 28.4)	25.0 (23.0; 28.7)	25.8 (23.3; 28.7)
Euro II Score	1.12 (0.85; 1.51)	1.19 (0.69; 2.45)	1.30 (0.88; 2.13)	1.24 (0.81; 2.07)
Mitral valve surgery	10 (41.7%)	15 (65.2%)	25 (53.2%)	50 (53.2%)
Aortic valve surgery	13 (54.2%)	9 (39.1%)	21 (44.7%)	43 (45.7%)

Characteristics of the different volatile and intravenous sedated patients. Abbreviations: BMI, body mass index; Euro II Score, European System for Cardiac Operative Risk Evaluation.

## Data Availability

The data cannot be shared publicly. The datasets generated and/or analyzed during the current study are not publicly available due to national data protection laws but are available upon reasonable request from the corresponding author or via the data protection officer of the University Hospital of Frankfurt (datenschutz@unimedizin-ffm.de).

## Abbreviations

The following abbreviations are used in this manuscript:

ACD	Anesthetic Conserving/Reflection Devices
HME	Heat moist exchanger
PRIS	Propofol-related infusion syndrome
RASS	Richmond Agitation and Sedation Score
